# *Mycobacterium tuberculosis* complex in wildlife: Review of current applications of antemortem and postmortem diagnosis

**DOI:** 10.14202/vetworld.2020.1822-1836

**Published:** 2020-09-09

**Authors:** Yusuf Madaki Lekko, Peck Toung Ooi, Sharina Omar, Mazlina Mazlan, Siti Zubaidah Ramanoon, Sabri Jasni, Faez Firdaus Abdullah Jesse, Azlan Che-Amat

**Affiliations:** 1 Department of Veterinary Clinical Studies, Faculty of Veterinary Medicine, Universiti Putra Malaysia, 43400 UPM Serdang, Selangor, Malaysia; 2 Department of Veterinary Medicine, Faculty of Veterinary Medicine, University of Maiduguri, 1069 PMB, Maiduguri, Borno State, Nigeria; 3 Department of Veterinary Pathology and Microbiology, Faculty of Veterinary Medicine, Universiti Putra Malaysia, 43400 UPM Serdang, Selangor, Malaysia; 4 Department of Farm and Exotic Animal Medicine and Surgery, Faculty of Veterinary Medicine, Universiti Putra Malaysia, 43400 UPM Serdang, Selangor, Malaysia; 5 Department of Paraclinical, Faculty of Veterinary Medicine, Universiti Malaysia Kelantan, Pengkalan Chepa, 16100 Kota Bharu, Kelantan, Malaysia

**Keywords:** culture, ELISA, gamma interferon test, genotyping, histopathology, *Mycobacterium tuberculosis* complex, polymerase chain reaction, wildlife

## Abstract

Tuberculosis (TB) is a chronic inflammatory and zoonotic disease caused by *Mycobacterium tuberculosis* complex (MTBC) members, which affects various domestic animals, wildlife, and humans. Some wild animals serve as reservoir hosts in the transmission and epidemiology of the disease. Therefore, the monitoring and surveillance of both wild and domestic hosts are critical for prevention and control strategies. For TB diagnosis, the single intradermal tuberculin test or the single comparative intradermal tuberculin test, and the gamma-interferon test, which is regarded as an ancillary test, are used. Postmortem examination can identify granulomatous lesions compatible with a diagnosis of TB. In contrast, smears of the lesions can be stained for acid-fast bacilli, and samples of the affected organs can be subjected to histopathological analyses. Culture is the gold standard test for isolating mycobacterial bacilli because it has high sensitivity and specificity compared with other methods. Serology for antibody detection allows the testing of many samples simply, rapidly, and inexpensively, and the protocol can be standardized in different laboratories. Molecular biological analyses are also applicable to trace the epidemiology of the disease. In conclusion, reviewing the various techniques used in MTBC diagnosis can help establish guidelines for researchers when choosing a particular diagnostic method depending on the situation at hand, be it disease outbreaks in wildlife or for epidemiological studies. This is because a good understanding of various diagnostic techniques will aid in monitoring and managing emerging pandemic threats of infectious diseases from wildlife and also preventing the potential spread of zoonotic TB to livestock and humans. This review aimed to provide up-to-date information on different techniques used for diagnosing TB at the interfaces between wildlife, livestock, and humans.

## Introduction

Tuberculosis (TB) is a reemerging and chronic infectious disease caused by *Mycobacterium tuberculosis* complex (MTBC); it affects livestock, wildlife, and humans [1]. The disease has a major economic impact on livestock production, due to costs associated with testing and slaughtering affected livestock. As such, bovine TB is subjected to control and eradication programs in some developing countries [1-4]. Studies have shown that one in every three people living today is infected with *Mycobacterium tuberculosis*, inducing increased morbidity and mortality in developing countries [5-9]. It was estimated that 1.2 million deaths from TB due to MTBC species and 143,000 new cases of zoonotic TB caused by *Mycobacterium bovis* occurred globally in the year 2018. However, only 16 countries reported the detection of *M. bovis* among pulmonary or extrapulmonary TB patients in 2018. Since 2007, TB has been the 10^th^ leading cause of death worldwide from a single infectious agent, ranking above HIV. Most cases in 2018 occurred in South East Asia (44%), followed by Africa (24%), the Western Pacific (18%), the Eastern Mediterranean (8.1%), the Americas (2.9%), and then Europe (2.6%) [10].

This review is intended to provide an up-to-date information of the antemortem and postmortem diagnostic techniques for TB at the interfaces between wildlife, livestock, and humans, that could help researchers and decision-makers to choose the most appropriate diagnostic protocol and to obtain a better understanding of their performance for effective TB surveillance and control strategies under different epidemiological situations in wild animals.

## MTBC

The members of MTBC are *M. tuberculosis*, *M. bovis*, *M. bovis* BCG (Bacillus, Calmette, and Guérin), *M. africanum*, *M. microti*, *M. mungi*, *M. canetti*, *M. caprae*, and *M. pinnipedii* [11-18]. Other members are *Oryx bacillus* [19], known as *M. orygis* [20], and the dassie bacillus [21]. The MTBC is recognized by 99.9% similarity at the nucleotide level, which resembles the 16S rRNA sequences [22].

## MTBC in Wildlife

Over 40 free-ranging wild animal species are known to be infected with MTBC bacterial species, causing the disease. Host species may be “maintenance hosts” (infection persists without input from other sources) or “spillover hosts” (infection persists as long as there is input from an external source) [23]. Tuberculosis has been isolated from different types of wildlife, such as badgers, coyotes, raccoons, hares, rabbits, hedgehogs, brushtail possums, coatis, capybaras, lions, deer, elk, wild boars, foxes, primates and pinniped (gray seal) [24-26]. The Eurasian wild boar acts as a reservoir for TB in the Iberian Peninsula [27-31]. In addition, spillover transmission of MTBC to other wildlife species and domestic livestock has been reported in South African buffalo [32], leopards, hyenas, large-spotted genets, warthogs, bushpigs, eland, wildebeest, common duiker, honey badger, impala, lechwe, lions, cheetahs, chacma baboons, and greater kudu [32-36].

Reports of TB in wildlife, mainly in Asian elephants from various countries in Southeast Asia, have been published. For example in Thailand, *M. tuberculosis* was isolated from four Asian elephants by bacterial isolation and sequencing [25,37]. The MTBC has also been reported in an Asian elephant in Malaysia [38,39]. Reports have also been published from Thailand in 2010 of a captive Malayan tapir with disseminated TB caused by *M. tuberculosis* and confirmed by molecular methods [40]. In the British Isles, the European badger is the dominant reservoir host of TB [41]; in Australia, feral pigs, fallow deer, and water buffalo are the reservoir hosts, causing spillover transmission to domestic goats and cattle; while in North America (Michigan, Mexico), the hosts are white-tailed deer, but in Canada, they are white-tailed deer, wood bison, and elk. In New Zealand, the brushtail possum is causing the transmission to deer, ferrets, and cattle [28]. Studies showed that the lesions in wild animals appeared to be similar to those in domestic animals. However, there are variations in terms of the location and size of lesions in different affected animals [42,43].

## Transmission

Several factors influence the transmission of TB, including the number of infected animals, susceptible animals, routes of infection, anatomical location and lesions of disease, the structure of tuberculous lesions, pathogen shedding, and the infective dose in each infection route [44]. Environmental contamination is important for MTBC transmission [45]. Direct oral/nasal transmission is the major infection route, while indirect transmission routes are considered to be less important [46]. The thoracic and abdominal lesions suggest that MTBC is transmitted directly oro-nasally and indirectly through the environment through shared watering and feeding areas [47-49]. Though the indirect route of transmission is important for interspecies transmission through shared resources [50] is making the TB transmission within same species of animals becomes significance [51,52]. Drinking water points are critical risk areas for MTBC transmission, especially in semi-arid areas where there is a scarcity of water, which leads to the daily gathering of animals at water points [53]. Following excretion, the bacteria must withstand the environmental conditions for them to be fully pathogenic before they can infect susceptible hosts by indirect transmission [54].

## Antemortem Diagnosis of MTBC (Cell-mediated Immune [CMI] Response)

For antemortem diagnosis, single intradermal tuberculin test (SITT), single comparative intradermal tuberculin test (SCITT), and gamma interferon test (IFN-ɣ), which is regarded as a subsidiary test, are used [55-58]. These different analyses have their own shortcomings with regard to the results produced [59], but it is well accepted that they complement one another, especially when used synergistically; SCITT is regarded as a better diagnostic tool with the high specificity and moderate sensitivity, whereas the IFN-ɣ test has higher sensitivity with the lower specificity [59-62]. Therefore, the two tests may not recognize a certain proportion of actually affected animals, leading to infection remaining in the animals [62-65]. The intradermal tuberculin test calculates dermal swelling basically due to CMI response 72 h after the introduction of purified protein derivative (PPD) in the skin of the neck or the caudal fold. The surface of the neck is more sensitive than the surface of the caudal fold; for this reason, more doses of PPD are required in the caudal fold region to increase it’s sensitivity like that of neck region [66]. At the same time, the SCITT test correlates with the response against bPPD and is applied in the cervical region to increase the specificity [55].

The *in vitro* IFN-γ assay involves two steps. In the first step, fresh blood samples are incubated to initiate the release of IFN-γ by sensitized T lymphocytes. The second stage requires separating the plasma from the blood and observing the IFN-γ in the plasma by an ELISA assay. A benefit of the IFN-γ assay is that the interpretation criteria are adjusted to fit local situations, such as the epidemiological conditions, TB prevalence, and the extent of the local bovine TB control program. The IFN-γ test has higher sensitivity than the skin test because the former detects TB disease in livestock as early as 14 days after infection [67] and 60-120 days faster than the SCITT test [68]. The most essential antigens used complementary to PPDs are the early secretory antigens (ESAT-6), culture filtrate protein 10 (CFP-10), and Rv3615c [69-73]. Moreover, the inclusion of Rv3020c increases the diagnostic sensitivity without affecting the specificity [74].

## Postmortem Diagnosis of MTBC

### Direct smears

For the rapid identification of MTBC infections, a direct smear is required for the detection of TB organisms from tissue samples with suspected infection followed by Ziehl–Neelsen (ZN) stain.. The basic principle of the stain is that MTBC available in the smear can take up an aryl methane dye, for example, carbol fuchsin, into the cell, resulting in acid-fast staining reaction, after treatment with a weak acid-alcohol solution. This is attributable to the mycolic acids that are present in the thick bacterial cell wall [75,76]. The detection of bacterial organisms from the tissues, feces, or other biological substances by this method is reduced because of few MTBC and possible contamination with other related organisms. Direct staining could not shed light on the members of the MTBC, producing the infection or show variation among viable and non-viable cells [76,77].

### Histopathology

In free-ranging wildlife, the diagnosis of *Mycobacterium* spp. relies on gross findings and histopathological analyses. One reliable method is histopathological analysis with hematoxylin and eosin staining [77]. This technique has high specificity and the additional advantage of producing results within a few days. A correlation of 94% was found between the results obtained using histopathological analysis and culture methods, regarding an agreement on the diagnosis of *M. bovis* infection in cattle [78].

### Bacterial culture

Culture is regarded as the gold standard technique for diagnosing MTBC, as described previously [79,80], given its high specificity. However, it can occasionally produce false-negative results, and its sensitivity has reach only as high as 80% [81,82]. Pre-treatment and processing procedures such as homogenization, decontamination, and concentration, as well as culture media that prevent the growth of contaminating bacteria, are required for the successful isolation of MTBC bacteria [83]. Critical factors for successful culture growth are the decontamination methods employed in the process, the type of decontaminant, and the concentration of decontaminant [84]. Decontaminants that are commonly used for this purpose are cetylpyridinium chloride and benzalkonium chloride [85], oxalic acid [86,87], sulfuric acid [88], and sodium hydroxide [85].

For the isolation of mycobacteria, three types of media are typically used. The first is egg-based media, such as Stonebrink’s medium and Lowenstein–Jensen, with either glycerol or pyruvate. The second is agar-based media, such as Middlebrook 7H10 and 7H11, and tuberculosis blood agar medium, B83. The third type is liquid media, such as Middlebrook 7H9. The use of solid and liquid media together should maximize the sensitivity, an approach that has been approved for the isolation of mycobacteria in laboratories for human TB diagnosis globally [89-92]. There are limitations in the use of solid media for the screening of mycobacteria, although improvements have been made regarding the detection time and recovery rate through the use of broth-based culture systems, such as BACTEC 460, the Mycobacteria Growth Indicator Tube (BACTEC MGIT 960), and the Versa TREK system [90].

### Serology or antibody detection test (humoral immune response)

Diagnosis based on an antibody is the most widely used approach for detecting TB in wild boar and pigs because of the maintenance of antibodies during transportation, storage, and handling, and the fact that the protocol can be standardized in different laboratories [93-97]. A widely used antigen is the isolated PPD from *M. bovis* (bovine PPD) [95,98,99]. However, new research has focused on improving its specificity through the use of different antigens, for example, MPB83, as described by García-Bocanegra [100,101], MPB70 [102], or CFP10/ESAT-6 [103,104]. Responses to MPB83 were identified at an earlier stage during experimental infections, with antibody responses arising 3-4 weeks after infection [105-108]. Moreover, antibodies directed at ESAT-6 and MPB70 were observed at 12 weeks [109] and 20 months after experimental infection, respectively, as reported by Fifis [110]. Hence, the highest and more rapid response to MPB83 protein proves that it is a better protein for use in producing diagnostic antigens for serological tests [55]. Several novel diagnostic tests explicitly designed to detect MTBC infection in wildlife have been established, such as the DPP (dual-path platform) WTB (MPB83, MPB70), DPP VetTB (MPB83, ESAT-6, CFP-10) [99], TB ELISA-VK^®^kit test (bPPD) [111], INgezim TB CROM Ab, INgezim Tuberculosis DR (based on MPB83), INgezim TB Porcine (based on MPB83 and MPB70) [112], and P22 ELISA (MPB70, MPB83, ESAT-6, CFP-10) [104,113].

## Molecular Diagnosis

### Multiplex PCR in the diagnosis of MTBC

Multiplex PCR methods produce a higher rate of the detection of MTBC. These techniques amplify two or more target genes in a reaction simultaneously [114,115]. A single target could end in a false-negative result, as a number of the targeted genes are not present in some MTBC strains [115,116]. The most efficient approach is one based on primers that amplify segments of the *IS6110* element, especially targeting the 123 bp and 245 bp fragments. Insertion sequences (IS) are mobile genetic elements that constitute a form of repetitive DNA in bacterial genomes. Insertion *IS6110* is an IS of 1361 bp that is not present in mycobacteria other than members of the MTBC [117]. Another PCR that produces excellent recognition of *M. bovis* isolates relies on the amplification of a 500 bp DNA segment, especially targeted for the 123 bp and 245 bp fragments inside the RvD 1Rv2031c genomic sequence [118,119]. Multiplex PCR has the advantage of being fast and accurate and can differentiate *M. bovis* from other members of MTBC by amplification of two DNA fragments [115,116,120].

### Digital PCR (dPCR) in the diagnosis of MTBC

Digital PCR (dPCR) was more accurate [121] while some reported that it was not susceptible to inhibitors [122] compared with real-time quantitative PCR (qPCR) and does not require a calibration curve needed to produce a qPCR copy number [123]. The high precision of PCR and theoretical accuracy have led to its use to measure reference materials [124,125] and to enable other molecular methods to be optimized [126]. Because of the lipid-rich cell wall and large GC-rich bacterial genome (4.4 MB) of MTBC members, makes diagnosis using other molecular methods difficult [127]. The dPCR was chosen to analyze this problematic genome and to quantify plasmid and MTBC gDNA with different PCR master-mixes, pairs of primers, and platforms of dPCR [128].

### DNA markers for the detection of MTBC

Some important DNA markers used in differentiating MTBC from non-tuberculous mycobacteria (NTM) in the diagnosis of TB include *rrs (16S rRNA)*, *ITS (16S-23S rRNA)*, *IS6110*, *groEL2 (hsp65)*, *dnaJ*, *fbpA (32 kDa protein)*, *MPT64 (MPB64)*, *devR*, *PPE24 (KS4)*, and *lepA* [129].

#### rrs (16S rRNA)

Studies have revealed that the 16S rRNA sequence can be used for the detection of mycobacteria, requiring fewer than 10,000 bacteria [130]. For species that are not easily characterized using normal biochemical methods, 16S rRNA can be used for identification, leading to the recognition of new species [131]. Nucleic acid amplification methods such as PCR, real-time PCR, and reverse cross-blot hybridization PCR of the 16S rRNA gene can be used to distinguish between MTBC and NTM members [132]. However, the use of the 16S rRNA gene alone is insufficient to differentiate MTBC and NTM groups because members of MTBC have 99.9% nucleotide resemblance and contain similar 16S rRNA sequences, and there is little difference genetically among NTM species [133,134].

#### ITS (16S-23S rRNA)

PCR amplification of the 16S-23S rRNA internal transcribed spacer (ITS) is used to recognize the genus *Mycobacterium*, which is applied to distinguish between MTBC and NTM based on the specific primer sequence [135]. This biomarker ITS 16S-23S rRNA is a spacer that distinguishes 16S and *23S rRN*A genes. Its size ranges from 270 to 360 bp among species, with greater sequence variation than the 16S rRNA [136]. ITS sequencing has a better ability to detect variation in species [137]. When the *IS6110* gene is lacking, sequence analysis of ITS helps in identifying MTBC members [138]. Slow growers have shorter ITS than fast growers, with its length correlating with the growth rate. The ITS sequence is found among MTBC, with the same sequence existing among *M. tuberculosis*, *M. bovis*, *M. bovis* BCG, *M. africanum*, and *M. microti* [139].

#### IS6110

Insertion sequences (ISs) are short mobile genetic DNA elements encoding proteins responsible for the transposition activity, which permit them to spread within the genome. In the bacterial genome, the insertion sequence causes deletions, duplications, and rearrangements due to changes in the genomic plasticity of mycobacterial species [140]. *IS6110* is the most available and easily characterized insertion element, which is found mainly in MTBC; it is used to distinguish MTBC from other mycobacteria. Several copies of this element are available at different positions in the genome, which facilitates better strain genotyping [141]. *IS6110-*restriction fragment length polymorphism (RFLP) has been widely used for epidemiological research on TB and has high discriminatory power, but is not suitable for species with less number of *IS6110* copies [141]. *IS6110* PCR and real-time PCR have shown high sensitivity (71-87.9%) and specificity (95-98%) compared with culture and acid-fast bacilli [142].

#### GroEL2 (hsp65)

The identification of all mycobacterial strains is achieved using the *hsp65* gene, which encodes a heat shock protein [143]. *hsp65*-PCR-RFLP can distinguish between mycobacteria and NTM [144], with a sensitivity of 100% and specificity of 93.1%, in contrast to culture and microscopy methods [145]. *hsp65* can be applied for specific recognition of NTM and can identify *M. avium complex* and *M. intracellulare* [146]. *hsp65* sequencing analysis is more accurate (88.9%) for identifying NTM than 16S rRNA (75.9%) [134].

#### dnaj

The *dnaj* gene encodes a cold-shock protein, which is a genus-specific gene that is amplified from almost all mycobacteria [147]. MTBC has similar *dnaj* sequences, while NTM has nucleotide substitutions at positions 1415 (A-C), 1417b (G-T/A/C), and 1442 (C-A), which are distinguished by the application of *dnaj*-PCR-RFLP [148] GENECUBE (Toyobo, Japan). This is an automated gene analyzer based on real-time PCR that targets the *dnaj* gene to detect the presence of MTBC and MAC [149]. When 56 species of mycobacteria were sequenced for *dnaj*, 16S rRNA, and hsp65 genes, mean sequence similarity levels were 80.4%, 96.6%, and 91.1%, respectively. This proved that *dnaj* has higher discriminatory power than 16S rRNA and hsp 65 genes and is useful for differentiating NTM [150].

#### fbpA (32 kDa protein)

*fbpA* is a secreted protein that is present in most MTBC strains but absent in non-MTBC strains [151]. Sequencing analysis has revealed that the *fbpA* gene is similar between MTBC groups, but has distinct sequences within MTBC species, such as *M. kansasii*, *M. gastri*, *M. gardone*, and *M. malmoense* [152]. This *fbpA* gene has better discriminatory power than the *hsp65* gene for identifying different NTM species [153].

#### MPT64 (MPB64)

The MPB64 gene is generally found in members of the MTBC and is used as an indicator in the diagnosis of paratuberculosis (PTB), TB meningitis, and extrapulmonary TB [154]. PCR using MPB64, has been shown to have sensitivity of 88% and specificity of 100% [155]. It can be used to diagnose TB in sputum, cerebrospinal fluid (CSF), ascitic fluid, and urine samples, but tends to give false-positive results in blood samples [156]. Studies have shown that MPB64 is complementary to *IS6110* PCR and has the capacity to decrease false-negative results in strains lacking the *IS6110* element [157]. Gene mutation is the major cause of false-negative results [158].

#### DevR

*devR*encodes the cytoplasmic response regulator *DevR*, which works together with the membrane-bound sensor kinase *DevS* to form *DevRS*. During latent infection in the host, *DevRS* is responsible for adaptation and dormancy [159]. Despite reports that shorter fragments of the *devR* gene significantly increased the sensitivity of TB diagnosis [160], the sensitivity of *devR* PCR assay is lower than that of MPB64 and *IS6110* [161].

#### PPE24 (KS4)

Some MTBC genes that have been less used for biomarker include the KS4 fragment of the Rv1753c gene, encoding the protein PPE. It has sensitivity of 98% and specificity of 98% in identifying MTBC, and cross-reacts with *M. fortuitum* and *M. xenopi* [129].

#### LepA

The Rv2404c gene encodes an elongation factor that is required for protein synthesis in MTBC members, but not in NTM [162]. It has been used as a target for internal amplification control to develop a real-time PCR assay detecting MTBC [163].

## Genotyping Techniques used in MTBC Diagnosis

Molecular typing uses genetic markers to search for sources of outbreaks, to detect the epidemic or pandemic spread of particular strains, or to reconstruct the evolutionary trends of a specific group of bacteria. The majority of the genotyping methods were developed in the 1990s. At this time, efforts were made toward standardizing typing protocols to increase the quality of epidemiological research [164]. Genotyping is divided into two methods: The whole-genome techniques and partial-genome typing of MTBC [165]. The whole-genome techniques have the benefits of using all of the organism’s available genetic information. Knowledge of the mutation rate is a critical factor determining the effectiveness of different genotyping methods. The mutation rate describes the frequency at which molecular fingerprint patterns change and is important for appropriate analysis of molecular data for epidemiological or phylogenetic research [166,167]. A higher mutation rate could result inthe high estimation of epidemiologically unrelated MTBC, which occurs at hypervariable loci [90,168].

## Whole-genome Typing of MTBC

### Restriction endonuclease analysis (REA)

This technique uses three restriction enzymes, namely, *Bst*EII, *Pvu*II, and *Bcl*I, for typing *M. bovis* isolates [169] and the joining of DNA strands of whole-genome DNA at specific nucleotide sequences. These restriction endonucleases digest their targets into several smaller segments, which are then differentiated by standard agarose gel electrophoresis. The developed segment patterns are visualized and compared with other isolates, enabling the differentiation of strains. The development of REA enables us to confirm whether domestic animals have become infected on farms or whether the infection was the result of the purchase of an initially diseased animal [170,171]. In some countries, this technique is used for epidemiological evaluation [172], but is yet to gain wide acceptance for this because of technical problems and difficulties in interpreting patterns. However, it is applicable to MTBC prevention and control programs.

### Pulsed-field gel electrophoresis

The technique of pulsed-field gel electrophoresis (PFGE) was initiated to bypassthe problem of analysis of excessive smaller number of DNA segments produced by REA. The PFGE process utilizes restriction enzymes that provide a smaller number of sections, which are very large, to be differentiated by standard agarose gel electrophoresis; but it is easily resolved using a pulsed electrical field. Initially, this approach was developed for *M. tuberculosis* isolates [173], but it was later employed for *M. bovis* and *M. bovis* BCG [174,175].

### Whole-genome sequencing (WGS)

Publication of the whole-genome sequences of *M. tuberculosis*, *M. bovis* and *M. bovis* BCG [176-178] is among the major scientific achievements in molecular tuberculosis research. Since its introduction, WGS has been used and has brought changes in genotyping by enabling the highest level of discrimination. WGS is a reliable method for forensic transmission and characterization analysis, allowing the assessment of genetic differences over time and the identification of transmission patterns at the individual level [179]. At present, WGS cannot be applied for regular diagnosis because of its cost and the need for a bioinformatic method. However, this ongoing progress is expected to increase our understanding of *M. bovis* infection and its effect on local epidemics.

### Whole-genome microarray

The introduction of whole-genome sequence data for different species of the MTBC has made it possible to apply microarray technology to compare strains, which has been particularly used to sequence reference strains [180]. The major objective of this technique is to perform comparative genomic hybridization, referred to as comparative genomics (DNA microarray), and for the comparison of gene expression, known as transcriptomics or expression profiling (RNA microarray). The designs for these different approaches are the same and do not depend on the initial products (DNA or RNA). PCR products that are open reading frame (ORF) genes of reference strains are applied on a solid material (e.g., glass slides), then the amplification of PCR products for DNA and reverse transcriptase for RNA is performed, followed by the application of fluorescent dyes that are then hybridized on the slides [181]. Subsequently, the spots produced are visualized with a fluorescence reader and analyzed using the software. Comparative genomics clarifies discrepancies among groups of MTBC, and the application of whole genomes has shed light on the pathogenesis, host adaptation [182], and virulence of different *M. bovis* strains [183]. It also helps to identify chromosomal polymorphisms as genetic markers for clonal complexes [184-186]. However, this method can be used only for research studies, rather than for regular diagnosis.

## Partial-genome Typing of MTBC

### Restriction fragment length polymorphism

Restriction fragment length polymorphism (RFLP) analysis utilizes the restriction enzyme *Pvu*II or *Alu*I, followed by southern blotting and gel electrophoresis of the fragmented isolated DNA onto a nitrocellulose or nylon filter [115]. RFLP based on *IS6110* is the genotyping method commonly applied for *M. tuberculosis* strains, which contain up to 20 copies of this IS [187]. However, this method is not easy to apply in different laboratories [188]. This technique also lacks discrimination for low-copy-number strains such as *M. bovis*; it is not frequently used for typing or routine diagnosis [164,189-191].

### Spoligotyping

Spoligotyping, also known as spacer oligonucleotide typing, is a rapid and robust technique that enables MTBC isolates to be typed at high throughput, with no need for DNA purification [192]. In a previous study [193], researchers were able to identify a clustered regularly interspaced palindromic repeat (CRISPR) region peculiar to the MTBC known as the direct repeat (DR) region. It is composed of multiple 36 bp DRs interspersed by unique sequences referred to as spacers, of 25-41 bp in length [194]; a DR and its adjacent spacer are called a direct variant repeat (DVR). The strains differ in terms of the number of DVRs; the presence or absence of the spacers is used for strain typing. While 104 spacer sequences are found in the DR region, not all exhibit sufficient polymorphisms that are important for strain typing [194,195]. The appropriate spoligotyping mehods involved the use of 43 spacers [192]. Two primers are utilized to target individual DRs and amplify the entire DR region with subsequent hybridization of fragments on a blotting membrane, which is joined by covalent binding to oligonucleotides and visualized through chemiluminescence. The membranes are prepared in the laboratory or obtained commercially from Ocimum Biosolution Ltd. (Hyderabad, India). This reverse line blot hybridization analysis provides results that are simple to save due to translation of the patterns into a binary code (0, absence of spacer; and 1, presence of spacers), and globally approved names for the spoligotype patterns, which are copied from spoligotype websites (http://www.Mbovis.org; http://www.pasteur-guadeloupe.fr/tb/bd_myco.html) [196,197].

### Variable number tandem repeats

Variable number tandem repeat (VNTR) typing has focused on genetic loci that are found in the entire genome; these loci contain an enormous amount of data of repeated sequences. Due to various polymorphisms in the copy number of the repeats, the tandem repeats were successfully used to fingerprint bacterial genomes [198]. VNTR typing, also called multilocus variable number tandem repeat analysis (MLVA), has several benefits, such as relatively low cost, simplicity in terms of use, and the production of unambiguous results [199]. VNTR typing relies on the PCR amplification of targeted loci with a particular primer pair and then gel electrophoresis. The application of automatic sequencers to check the correct size of the amplified fragments has been optimized in several research and diagnostic laboratories, enabling automated high-impact genotyping [200]. For the interpretation of results, the application of an allele calling table related to several band sizes that are equivalent to the number of repeats in each locus is indispensable. The earlier VNTR locus found in the *M. tuberculosis* genome contains about 75 bp tandem repeats situated in the large ORF [201]. It was previously reported [202] that some loci are found in ORFs, for example, QUB11a, QUB18, QUB23, and QUB26. Many loci found within coding regions have repeat sizes that are multiples of three. Several scattered repeats are found in intergenic regions and are known to play an important role in the bacterial genome. In a previous study [203], these loci were called mycobacterial interspersed repetitive units (MIRUs), with a size range from 46 to 101 bp. VNTR typing of MTBC is also called MIRU-VNTR typing. The number of MIRUs per genome is around 40-50. VNTR typing is useful for epidemiological studies and can be used to recognize multiple infections [204,205].

### *IS6110* ample printing

Ample printing uses a polymorphic tandem repeat (MPTR) sequence [206], which is similar to the DR region comprising 10 bp direct repeats separated by 5 bp unique spacers. MPTR was found in atypical mycobacteria and possessed few polymorphisms in the MTBC. Nonetheless, for *M. tuberculosis*, excellent results have been obtained by applying this approach in combination with *IS6110*, referred to as *IS6110*-ample printing, which makes use of the gap between *IS6110* and copies of MPTR sequences. Because the results of ample printing differ significantly in terms of discrimination and reproducibility, it is not regularly used as a diagnostic technique [207-209].

### Random amplified polymorphic deoxyribonucleic acid (RAPD) analysis

Because of its simplicity, RAPD is usually used for genotyping organisms, as a PCR-based fingerprinting method [210]. It was initially established for the typing of *M. tuberculosis* isolates, as described previously [211]. However, it is rarely used for *M. bovis* because of poor results in terms of discrimination ability [207,212]. Problems associated with reproducibility have made this method unpopular for the genotyping of mycobacterial species.

### Multilocus sequence typing (MLST)

The primary purpose of MLST is to categorize organisms in terms of the level of neutral sequence diversity [213]. It has been applied to the characterization of organisms of bacterial origin by a technique of sequencing internal fragments of about 450-500 bp of approximately seven housekeeping genes. For each individual gene, differentsequences are allocated specific alleles, with the aim that an isolate with allelic data or sequence type (ST) is gained by joining the alleles at each locus. Housekeeping gene produce differences slowly, which makes them selectively neutral. Incontrast with other bacterial organisms with variation in housekeeping genes, members of MTBC are monomorphic with reduced discrimination levels [214] to improve discrimination, characterization of housekeeping genes such as encoding antigens and antibiotic-resistance genes, and genes targeting the region of insertion is necessary [215].

### RD typing

Region of Deletion (RD) typing is used for the differentiation of species that are members of MTBC. RD9is used to differentiate *M. tuberculosis* from other members of the MTBC and RD4 which is not found in all of *M. bovis* isolates. As a result, RDs were used as PCR targets as a rapid method of species identification; more recently, additional RDs were discovered, namely, RD2seal [216], RD1mic [217], RD1das [218], RD1 mun RD12oryx, and RD1 BCG, to distinguish *M. pinnipedii*, *M. microti*, *M. dassie bacillus*, *M. mungi*, *M. orygis*, and *M. bovis* BCG, respectively, from other species [18,20,219]. For PCR application, different methods are used, such as using three primers, involving two flanking and one internal primer [220,221], or four primers, involving two flanking and two internal primer pairs [137], to locate the presence or absence of the RDs. Several applications of PCR to find different RDs have been approved for species differentiation [222,223]. Because of the one-directional evolution of the RDs [224], these markers are beneficial for the emergence of MTBC and to find clonal complexes within the MTBC host-adapted members [185,186].

### Single-nucleotide polymorphism (SNP)

SNP typing is an essential form of genotyping analysis that enables differentiation among members of MTBC, for example, *M. bovis* [16] and *M. caprae* [225]. A previous study [163] described a unique *M. caprae-*specific SNP in the *M. tuberculosis* H37Rv *lepA* (*Rv2404c*) gene, which is particular to bovine and cervidae isolates [162]. Genotyping using SNP serves as an excellent technique for the recognition of specific lineages in *M. tuberculosis* [226,227] and *M. bovis* [228,229]. A commercial DNA stripuses the RD1 deletion for the identification of *M. bovis* BCG and differentiation of MTBC species [230], which makes it a useful tool for the diagnosis of tuberculosis in different wild animals [34-36].

## Conclusion

Tuberculosis remains a global threat at the livestock – wildlife – human interface which require different diagnostic approach in the disease detection, prevention and control strategies. Each of these diagnostic methods has its own merits and demerits in terms of application and efficiency. The antemortem diagnosis detected CMI responses using the SITT, SCITT, and gamma interferon test (IFN-ɣ). The skin test requires animals to be handled twice, which is dangerous, especially for some wildlife, and the test cannot be repeated for about 90 days due to immunological sensitization to the PPD antigens. The higher sensitivity of the IFN-γ test relative to the skin test is because the IFN-γ test detects TB disease in animals as early as 14 days after infection and 60-120 days faster than the SCITT test. For postmortem diagnosis, direct smears are the fastest, cheapest, and simplest way to detect acid-fast MTBC. For this reason, a direct smear of tissue samples can be stained following the Ziehl–Neelsen technique to provide a presumptive identification of MTBC. Histopathological techniques have high specificity and the advantage of producing results within a few days. A correlation was found between the results obatained using histopathology and culture methods. Serology or antibody detection is the most widely used approach for the detection of TB in wild animals because of the maintenance of antibodies during transportation, storage, and handling, the ability to standardize the protocol in different laboratories, and the fact that the results are obtained within a short period of time. One disadvantage of antibody detection is that humoral responses to infection occur at the later stage of the disease. Bacterial culture remains the gold standard diagnostic method for MTBC, but requires several weeks to obtain positive results due to the extremely fastidious growth of tuberculous mycobacteria. It is used for species differentiation and has high sensitivity and specificity. Molecular diagnostic techniques of MTBC have advantages over conventional methods due to their sensitivity and specificity, being less complicated, less time-consuming, but expensive and not cost-effective compared with conventional methods for the early confirmation of the diagnosis of TB in both humans and animals. Molecular genotyping methods are used to differentiate between MTBC and NTM, and for species differentiation, which are not possible by the conventional approach. The combination of antemortem, postmortem, and molecular diagnostic techniques is the best option for controlling and preventing TB in both humans and wild animals.

## Authors’ Contributions

YML and AC conceived and designed the review outlines, collected the data and analyzed the data. PTO, SO, MM, SZR, SJ, and FFAJ analyzed the data and supervised the writing of the manuscripts. All authors have read and approved the final manuscript.
